# *In vivo* investigation of hyperpolarized [1,3-^13^C_2_]acetoacetate as a metabolic probe in normal brain and in glioma

**DOI:** 10.1038/s41598-019-39677-2

**Published:** 2019-03-04

**Authors:** Chloé Najac, Marina Radoul, Lydia M. Le Page, Georgios Batsios, Elavarasan Subramani, Pavithra Viswanath, Anne Marie Gillespie, Sabrina M. Ronen

**Affiliations:** 1 Department of Radiology and Biomedical Imaging, University of California San Francisco, San Francisco, CA United States; 2 Department of Physical Therapy and Rehabilitation Science, University of California San Francisco, San Francisco, CA United States

## Abstract

Dysregulation in NAD^+^/NADH levels is associated with increased cell division and elevated levels of reactive oxygen species in rapidly proliferating cancer cells. Conversion of the ketone body acetoacetate (AcAc) to β-hydroxybutyrate (β-HB) by the mitochondrial enzyme β-hydroxybutyrate dehydrogenase (BDH) depends upon NADH availability. The β-HB-to-AcAc ratio is therefore expected to reflect mitochondrial redox. Previous studies reported the potential of hyperpolarized ^13^C-AcAc to monitor mitochondrial redox in cells, perfused organs and *in vivo*. However, the ability of hyperpolarized ^13^C-AcAc to cross the blood brain barrier (BBB) and its potential to monitor brain metabolism remained unknown. Our goal was to assess the value of hyperpolarized [1,3-^13^C_2_]AcAc in healthy and tumor-bearing mice *in vivo*. Following hyperpolarized [1,3-^13^C_2_]AcAc injection, production of [1,3-^13^C_2_]β-HB was detected in normal and tumor-bearing mice. Significantly higher levels of [1-^13^C]AcAc and lower [1-^13^C]β-HB-to-[1-^13^C]AcAc ratios were observed in tumor-bearing mice. These results were consistent with decreased BDH activity in tumors and associated with increased total cellular NAD^+^/NADH. Our study confirmed that AcAc crosses the BBB and can be used for monitoring metabolism in the brain. It highlights the potential of AcAc for future clinical translation and its potential utility for monitoring metabolic changes associated with glioma, and other neurological disorders.

## Introduction

Glioma are the most common type of brain tumors, accounting for up to 30% of all brain tumors and 80% of all malignant tumors^1^. They are categorized into three types (astrocytoma, oligodendroglioma and glioblastoma) and four World Health Organization grades (I to IV) based on clinical criteria as well as histological and molecular features^1–4^. Of all glioma, glioblastoma (GBM, Grade IV or high-grade) are the most frequent, aggressive and lethal brain tumors, with a median survival rate as low as 15 months^1^. Grade II and III glioma, also referred to as low-grade glioma (LGG), have a significantly better prognosis than GBM but survival still remains less than 7 years^2,5^. Current standard of care for all brain tumors is a combination of treatments and includes surgical resection, radiation therapy and chemotherapy^4^. However, currently, no treatment can fully cure glioma. Therefore, new therapies and clinically translatable methods to detect tumors and monitor response, are urgently needed to improve patient survival and quality of life.

Independent of their grade, glioma are associated with a broad rewiring of the metabolic pathways allowing the tumor to survive and grow^6–8^. In particular, reactive oxygen species (ROS) are intracellular chemical species and byproducts of oxygen metabolism that regulate cell signaling and homeostasis. Elevated ROS levels play an important role in tumor development and are associated with increased rates of cell division^6,8,9^. The increase in ROS in cancer cells activates signaling pathways and transcription factors promoting tumorigenesis. Subsequently, cancer cells increase their level of antioxidants to prevent cell death and thus allow cancer progression^6,8,9^. NADPH plays a major role in controlling cellular redox, regulating glutathione reduction and the thioredoxin system^10^. In LGG, NADPH also plays an important role in tumor development^11–14^. Specifically, up to 70% of LGG and 90% of secondary upgraded GBM harbor a heterozygous missense mutation in one copy of the gene encoding for the isocitrate dehydrogenase 1 (IDH1) enzyme, and this mutation is now recognized as an essential driver of LGG development^11–13^. Mutant IDH1 catalyzes the conversion of α-ketoglutarate (α-KG) to 2-hydroxyglutarate (2-HG) and NADPH serves as a co-factor for this reaction leading to changes in redox. The mutation results in decreased NADPH and glutathione levels and therefore increased ROS levels^15^. Importantly, NADH and NAD^+^ also play a critical role in controlling cellular redox through several pathways, with some studies reporting a direct antioxidant role of NADH and the NAD^+^-mediated inhibition of ROS^10^. Furthermore, NAD^+^ kinase allows conversion of NAD^+^ to NADP^+^ and nicotinamide nucleotide transhydrogenase transfers reducing equivalents from NADH to NADPH. Therefore, the maintenance of both NAD^+^/NADH and NADP^+^/NADPH levels is highly interconnected^10^. Considering the importance of redox in cancer in general, and in the development of LGG in particular, imaging methods that could shed light on redox and associated metabolic reactions could help in the non-invasive characterization of glioma.

Several optical methods, using fluorescence or multi-photon microscopy, have been used to measure redox *in vivo*^16,17^. ^31^P magnetic resonance spectroscopy (MRS) was also recently used to measure the intracellular NAD^+^/NADH ratio in the healthy human brain and age-dependent changes^18,19^. A promising approach is the use of ^13^C MRS combined with hyperpolarized metabolites that are metabolized in a redox-dependent manner. Indeed, as recently reviewed by us and others, dissolution dynamic nuclear polarization (DNP) leads to a more than 10,000-fold enhancement in the signal to noise ratio (SNR) of metabolites and provides a tool to monitor metabolism in real time^20,21^. Over the past decade, several hyperpolarized ^13^C agents have been developed and applied to the imaging of normal and diseased tissues^21^. The most common probe, hyperpolarized [1-^13^C]pyruvate, is currently in clinical trials for patients with prostate cancer, brain tumors, liver metastases and heart disease^22,23^. To assess cellular redox, previous studies have shown the utility of the hyperpolarized dehydroascorbate (DHA)/Vitamin C redox pair in healthy liver, kidney, brain and cancer mouse models^24,25^. However, the toxicity of DHA is likely to limit its clinical translation^25^. Another redox probe recently reported is hyperpolarized ^13^C-acetoacetate (^13^C-AcAc), which was used to assess mitochondrial redox in lymphoma cells, perfused rat hearts under ischemia, rat hearts *in vivo* in fed and fasted conditions, and rat kidneys *in vivo*^26–30^. This approach is based on the fact that the reversible interconversion of AcAc and β-hydroxybutyrate (β-HB) depends on the availability of NADH and the β-hydroxybutyrate dehydrogenase enzyme (BDH), which is located on the inner mitochondrial membrane. As such, monitoring the fate of AcAc can serve to assess redox, and previous studies indicate that the ratio of acetoacetate-to-β-hydroxybutyrate (AcAc-to-β-HB) is associated with mitochondrial redox status (NAD^+^/NADH)^31,32^. Importantly for brain studies, AcAc and β-HB are two ketone bodies that serve as the brain’s major alternative fuel to glucose, and are transported through the blood brain barrier (BBB), cell and mitochondrial membranes via the monocarboxylate transporters (MCT1 and 2)^32,33^. In addition, previous studies show that BDH expression is lower in glioma compared to normal brain, pointing to the potential utility of monitoring the fate of hyperpolarized AcAc^34,35^. Finally, previous work demonstrated that ^13^C-AcAc fulfills the technical requirements for a useful hyperpolarized probe: its polarization enhancement, which represents the efficiency of the dissolution DNP method, and the longitudinal T_1_ relaxation, that characterizes the lifetime of the hyperpolarized signal, are both sufficient to monitor metabolism^27,28,30^. However, to date, we have found no reports of the use of hyperpolarized ^13^C-AcAc in the brain.

The goal of our study was therefore to evaluate the feasibility of using hyperpolarized ^13^C-AcAc *in vivo* in the brain. We first confirmed that production of hyperpolarized ^13^C-β-HB can be detected in the healthy mouse brain following intravenous injection of hyperpolarized ^13^C-AcAc. Then, we show that the ratio of hyperpolarized ^13^C-β-HB-to-AcAc is lower in tumor-bearing mice when compared to normal controls and that this metabolic difference is associated with an increase in total cellular NAD^+^/NADH (mitochondrial plus cytosolic) ratio and a decrease in BDH activity in tumors compared to normal brain tissue.

## Results

### Characterization of hyperpolarized [1,3-^13^C_2_]acetoacetate

**Figure 1:**
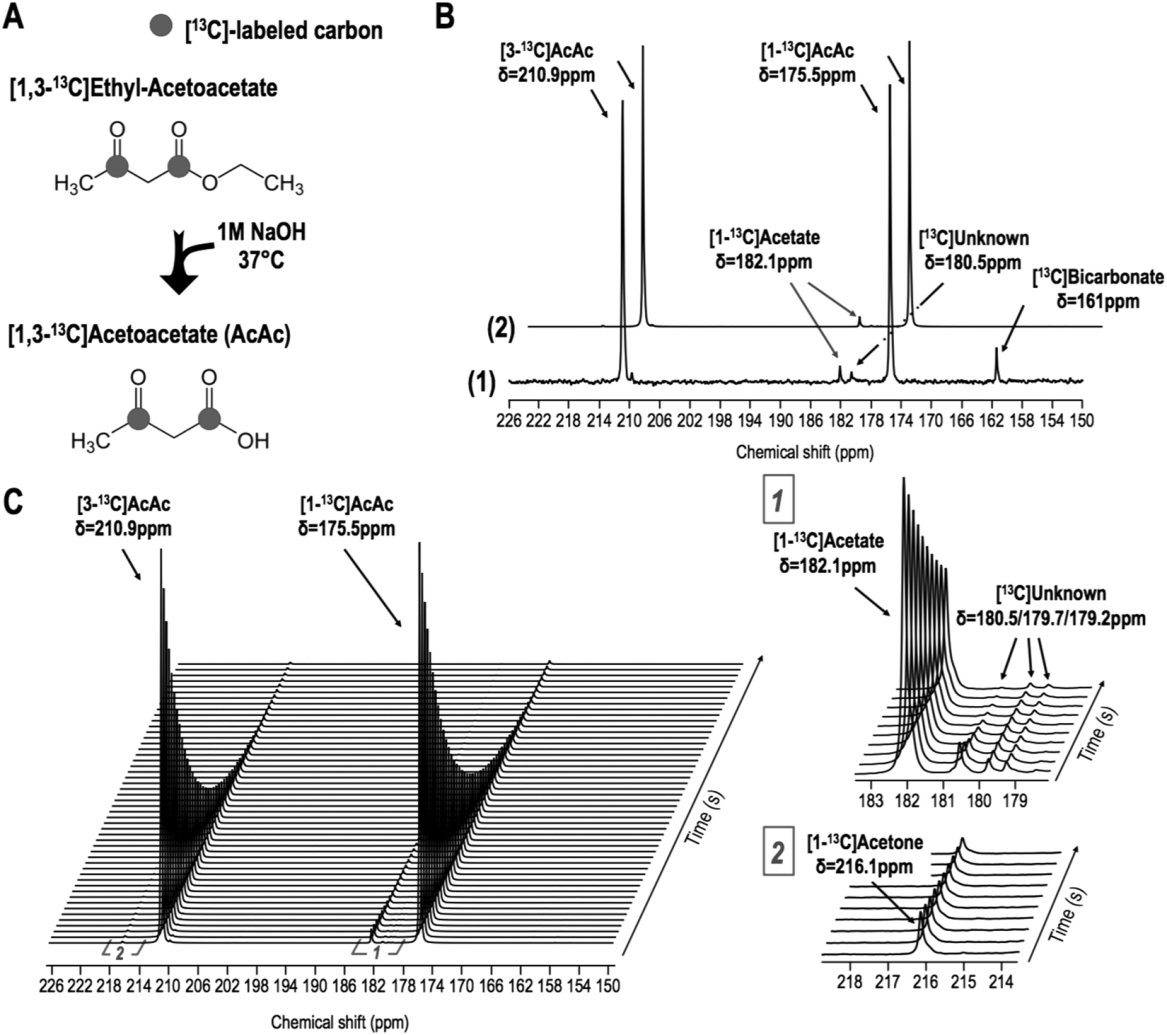
Characterization of the hyperpolarized probe, [1,3-^13^C_2_]acetoacetate ([1,3-^13^C_2_]AcAc). (**A**) [1,3-^13^C_2_]AcAc was prepared by mixing 250 μL of [1,3-^13^C_2_]ethyl-AcAc with 4 mL of 1 M NaOH at 37 °C for 24 hours. (**B**) [1,3-^13^C_2_]AcAc thermal equilibrium spectrum (1, NT = 16, x80) and first hyperpolarized spectrum (2, NT = 1) were acquired at 11.7T and showed a ~20% increase in liquid-state polarization level by the dissolution dynamic nuclear polarization technique (NT = number of transient). Resonances of [1-^13^C]AcAc (δ[1-^13^C]AcAc = 175.5 ppm), [3-^13^C]AcAc (δ[3-^13^C]AcAc = 210.9 ppm) and ^13^C-contaminants (δ[1-^13^C]Acetate = 182.1 ppm, δ[^13^C]Unknown = 180.5 ppm and δ[^13^C]Bicarbonate = 161 ppm) were detectable. (**C**) Stack plot of ^13^C MR spectra of hyperpolarized [1,3-^13^C_2_]AcAc in solution acquired at 11.7T showing decay of the hyperpolarized signals as a function of time (temporal resolution 3 sec). Resonances of [1-^13^C]AcAc (δ[1-^13^C]AcAc = 175.5 ppm), [3-^13^C]AcAc (δ[3-^13^C]AcAc = 210.9 ppm) and ^13^C-contaminants (δ[1-^13^C]Acetate = 182.1 ppm, δ[^13^C]Unknown = 180.5, 179.7, 179.2 ppm and δ[1-^13^C]Acetone = 216.1 ppm) were detectable.

[1,3-^13^C_2_]acetoacetate ([1,3-^13^C_2_]AcAc) was synthesized by hydrolyzing its ethyl ester with sodium hydroxide (Fig. 1A). It was then dissolved in 1:3 water:dimethylsulfoxide with trityl radical OX063 (Oxford Instruments, UK) and gadolinium. Following polarization, resonances of [1-^13^C]AcAc and [3-^13^C]AcAc were detected with an enhancement of 18 ± 5% (n = 3) and 22 ± 4% (n = 3), respectively, when compared to the thermal spectrum (Fig. 1B and Table S1). T_1_ values of hyperpolarized [1-^13^C]AcAc and [3-^13^C]AcAc were measured in solution at 11.7T by acquiring dynamic data with a short TR and a small flip angle and fitting the decay of hyperpolarized signal using a mono-exponential curve (Fig. 1C). Similar to previous reports^28,36^, the T_1_ values of hyperpolarized [1-^13^C]AcAc and [3-^13^C]AcAc were 31 ± 3 sec (n = 5) and 27 ± 1 sec (n = 5), respectively (Table S1). Additional resonances, attributed to [1-^13^C]acetone (δ[1-^13^C]acetone = 216.1 ppm), [1-^13^C]acetate (δ[1-^13^C]acetate = 182.1 ppm), unknown contaminants (δ[^13^C]unknown = 180.5, 179.7 and 179.2 ppm) and [^13^C]bicarbonate (δ[^13^C]bicarbonate = 161 ppm) were detectable on thermal and hyperpolarized data (Fig. 1B,C). Importantly, however, the contaminants do not resonate at the frequency of expected metabolic products of interest, i.e. [1-^13^C]β-HB (δ[1-^13^C]β-HB = 181.1 ppm) and [3-^13^C]β-HB (δ[3-^13^C]β-HB = 66.5 ppm).

### *In vivo* hyperpolarized [1,3-^13^C_2_]acetoacetate in the healthy and tumor-bearing mouse brain at 14.1T

**Figure 2:**
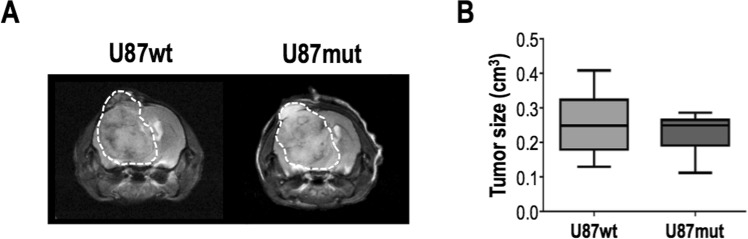
Tumor size quantification. (**A**) Representative T_2_-weighted images acquired on a 14.1T MRI scanner of U87wt (left) and U87mut (right) tumor-bearing mice and (**B**) corresponding tumor size quantification at time of the hyperpolarized experiments.

**Figure 3:**
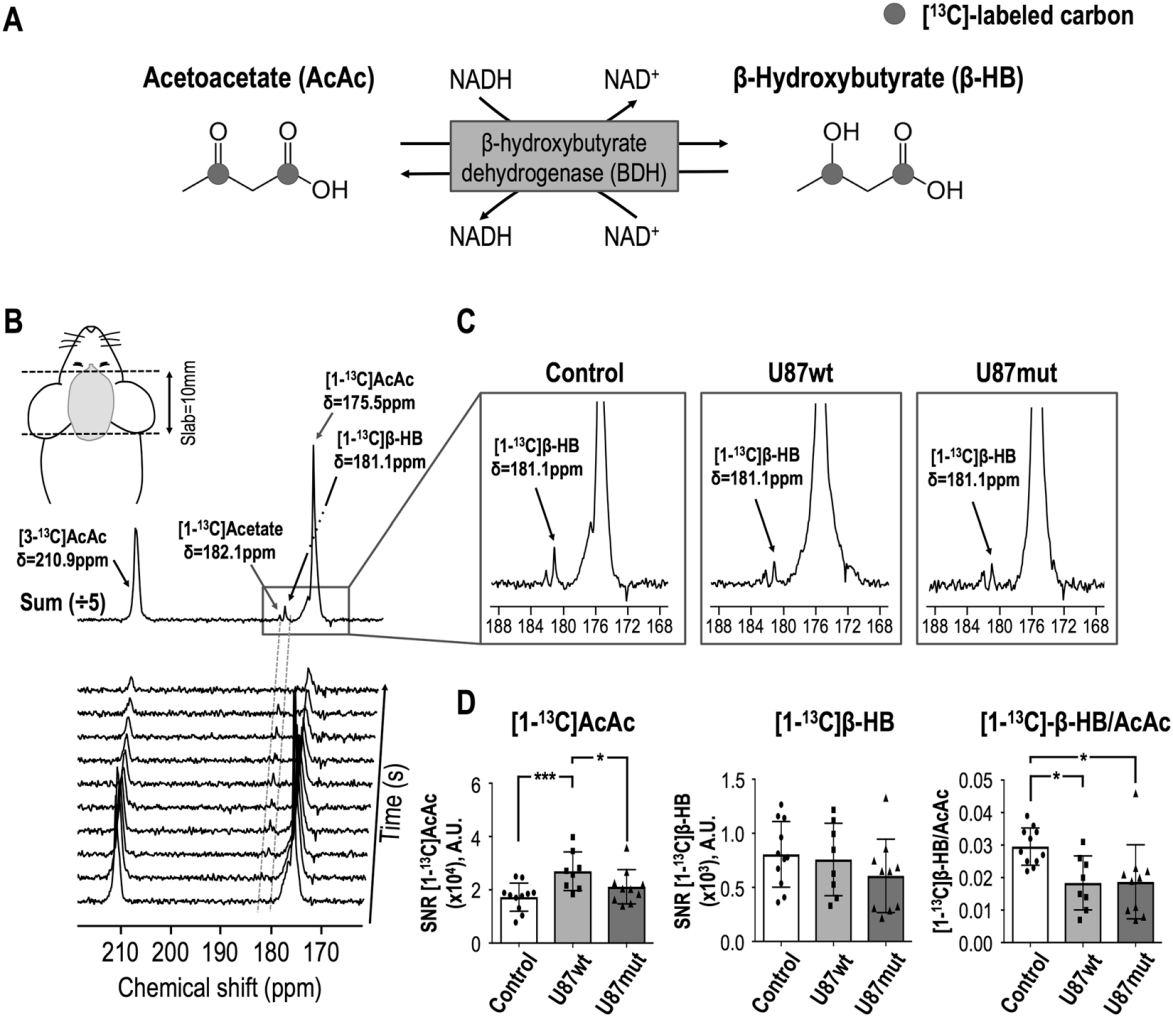
Representative hyperpolarized ^13^C data from 1D slab dynamic acquisitions acquired on a 14.1T MRI scanner. (**A**) [1,3-^13^C_2_]AcAc metabolism, showing conversion into [1,3-^13^C_2_]β-HB mediated by the β-hydroxybutyrate dehydrogenase (BDH) enzyme with NADH as a co-factor. (**B**) Stack plot of hyperpolarized ^13^C data acquired from a 1D 10 mm-thick slab (FA = 20°) covering the whole brain of a control mouse (tumor-free), showing decay of hyperpolarized [3-^13^C]AcAc (δ[3-^13^C]AcAc = 210.9 ppm) and [1-^13^C]AcAc (δ[1-^13^C]AcAc = 175.5 ppm) and production of hyperpolarized [1-^13^C]β-HB (δ[1-^13^C]β-HB = 181.1 ppm) as a function of time (time resolution 4 sec). Hyperpolarized acquisitions were started 10 sec after the beginning of the hyperpolarized injection (350 μL of [1,3-^13^C_2_]AcAc injected over 12 sec). All dynamic data were summed. Resonances of [1-^13^C]AcAc and [1-^13^C]β-HB were fitted with a Lorentzian-Gaussian line shape using MestreNova for each animal and integrals of the fits normalized to SD of the noise were quantified. (**C**) Representative hyperpolarized ^13^C data obtained from the sum of all dynamic data from a control mouse (left), U87wt-bearing mouse (middle) and U87mut-bearing mouse (right), showing that hyperpolarized [1-^13^C]β-HB production could be detected in all three groups. (**D**) Quantification of [1-^13^C]AcAc and [1-^13^C]β-HB levels and ratio of [1-^13^C]β-HB-to-[1-^13^C]AcAc. A significant increase in [1-^13^C]AcAc level in U87wt tumor-bearing mice and control mice and between U87wt and U87mut tumor-bearing mice was observed. A significant decrease in [1-^13^C]β-HB-to-[1-^13^C]AcAc ratio in both tumor models compared to normal mice was detected. No difference between U87wt and U87mut tumor-bearing mice was observed. SNR, signal to noise ratio; A.U., arbitrary units; AcAc, acetoacetate; β-HB, β-hydroxybutyrate.

**Figure 4:**
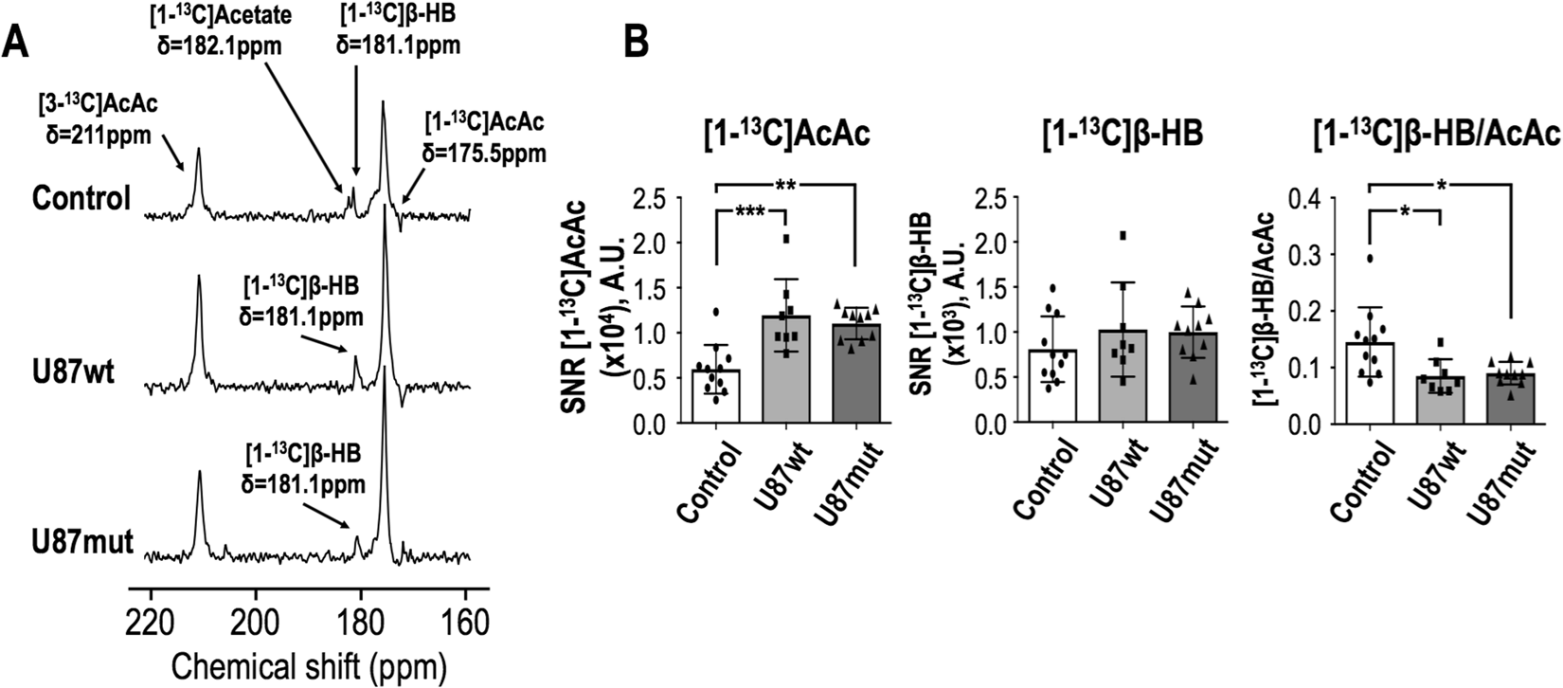
Representative hyperpolarized ^13^C data from 1D slab 90° acquisitions acquired on a 14.1T MRI scanner. (**A**) Representative hyperpolarized ^13^C data acquired from a 1D 10 mm-thick slab (FA = 90°) in a control mouse (top), U87wt-bearing mouse (middle) and U87mut-bearing mouse (bottom). Signals from hyperpolarized [3-^13^C]AcAc (δ[3-^13^C]AcAc = 210.9 ppm), [1-^13^C]AcAc (δ[1-^13^C]AcAc = 175.5 ppm) and [1-^13^C]β-HB (δ[1-^13^C]β-HB = 181.1 ppm) could be detected in all three groups. Resonances of [1-^13^C]AcAc and [1-^13^C]β-HB were fitted with a Lorentzian-Gaussian line shape using MestreNova for each animal and integrals of the fits normalized to SD of the noise were quantified. (**B**) Quantification of [1-^13^C]AcAc and [1-^13^C]β-HB levels and ratio of [1-^13^C]β-HB-to-[1-^13^C]AcAc showed a significant increase in [1-^13^C]AcAc level and a significant decrease in [1-^13^C]β-HB-to-[1-^13^C]AcAc ratio in both tumor models compared to normal mice. No difference between U87wt and U87mut tumor-bearing mice was observed. SNR, signal to noise ratio; A.U., arbitrary units; AcAc, acetoacetate; β-HB, β-hydroxybutyrate.

Hyperpolarized acquisitions were performed on tumor-bearing animals (U87wt and U87mut mice) when tumors reached a volume of ~0.24 cm^3^ (Fig. 2). Signals from hyperpolarized [1-^13^C]AcAc, [3-^13^C]AcAc and [1-^13^C]β-HB could be detected in tumor-bearing and tumor-free animals in hyperpolarized ^13^C dynamic and 90° acquisitions (Fig. 3B,C). However, signal from [3-^13^C]β-HB could not be detected in all groups due to its shorter T_1_ value. As illustrated in Fig. 3D, summed dynamic data showed a significantly higher SNR for [1-^13^C]AcAc in U87wt-bearing mice (n = 8) compared to controls (n = 11) and U87mut-bearing mice (n = 10), but no significant differences in the SNR of [1-^13^C]-β-HB between any of the groups (*p* value  > 0.35). The ratio of [1-^13^C]β-HB-to-[1-^13^C]AcAc was significantly lower in U87wt- and U87mut-bearing mice compared to controls. Similar to dynamic data, results from 90° acquisitions also showed a significantly higher SNR for [1-^13^C]AcAc and lower ratio of [1-^13^C]β-HB-to-[1-^13^C]AcAc in U87wt- (n = 8) and U87mut-bearing (n = 10) mice compared to controls (n = 11) but no differences in the SNR of [1-^13^C]-β-HB between all groups (*p* value > 0.46, Fig. 4A,B). Quantification of signal from [3-^13^C]-AcAc showed similar results as signal from [1-^13^C]AcAc (Fig. S2).

### Evaluation of NAD^+^/NADH ratio and BDH activity in tissues

**Figure 5:**
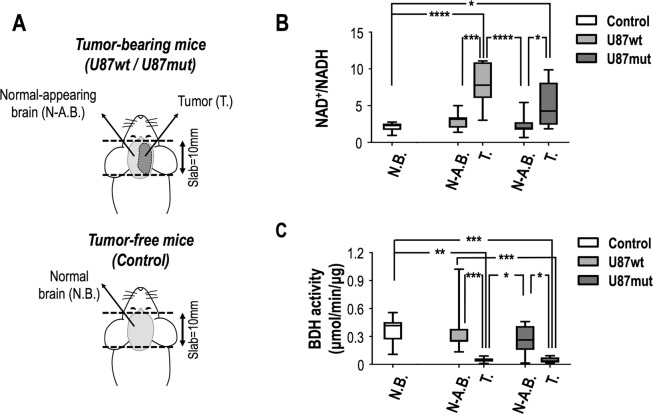
NAD^+^/NADH ratio and BDH activity quantified by spectrophotometric assays. (**A**) 1D slab ^13^C acquisitions were 10 mm-thick and therefore covered the whole brain. In tumor-bearing mice, the slab contained both tumor and normal-appearing brain whereas in tumor-free (control) mice, it contained only normal brain. At sacrifice, both normal-appearing and tumor tissues from tumor-bearing mice and normal brain tissues from control mice were freeze-clamped and kept at −80 °C for spectrophotometric assays. (**B**) NAD^+^ and NADH levels were measured and NAD^+^/NADH ratios were quantified. No differences were observed in NAD^+^/NADH ratio between control normal brain tissue and tumor-bearing mouse normal-appearing brain tissue and between U87wt and U87mut tumor tissue. A significant increase in NAD^+^/NADH ratio was observed between controls normal brain and U87wt/U87mut tumor tissues as well as between tumor-bearing normal-appearing brain tissues and U87wt/U87mut tumor tissues. (**C**) BDH activity from isolated mitochondria was quantified. No differences were observed between controls normal brain tissues and tumor-bearing mice normal-appearing brain tissues. A significant decrease in BDH activity in tumor tissues compared to normal-appearing brain tissues was observed for both tumor models. A significant decrease was measured between U87wt and U87mut tumor tissues and normal brain tissues from healthy controls. N.B., Normal Brain; N-A.B., Normal-Appearing Brain; T., Tumor.

The enzymatic conversion of acetoacetate to β-hydroxybutyrate requires NADH as a co-factor and relies on BDH enzyme activity. To assess if changes in the *in vivo* ratio of [1-^13^C]β-HB-to-[1-^13^C]AcAc could be linked to alterations in cellular redox, we used a commercial spectrophotometric assay to evaluate the levels of NAD^+^ and NADH. The hyperpolarized ^13^C slab from which data were acquired contained both tumor and normal-appearing brain tissue for tumor-bearing mice, but only normal brain tissue for tumor-free mice. Therefore, tumor and normal-appearing brain tissues were collected and freeze-clamped for tumor-bearing mice, while only normal brain samples were collected for tumor-free mice (Fig. 5A). The NAD^+^/NADH ratios in U87wt tumor samples (n = 7), U87mut (n = 10) tumor samples, normal-appearing brain tissue from both tumor groups, and tissue from control tumor-free mice (n = 8) were, respectively, 7.8 ± 2.8, 5.1 ± 3.1, 2.9 ± 1.2, 2.3 ± 1.3 and 2.1 ± 0.6 (Fig. 5B). The NAD^+^/NADH ratio was therefore significantly higher in the tumor tissue compared to normal-appearing brain tissue in both U87wt- and U87-mut bearing mice as well as compared to normal brain tissue from control mice. We then investigated BDH enzyme activity in order to determine if changes in redox and conversion of hyperpolarized [1-^13^C]AcAc to [1-^13^C]β-HB in our tumor models could also be explained by alterations in the BDH enzyme. BDH enzyme activities in U87wt tumor samples (n = 7), U87mut (n = 8) tumor samples, normal-appearing brain tissue samples and tissue from controls (n = 7) were 0.05 ± 0.03, 0.37 ± 0.30, 0.04 ± 0.03, 0.27 ± 0.16 and 0.36 ± 0.15 μmol/min/μg of protein respectively (Fig. 5C), thus BDH enzyme activity was significantly lower in tumors compared to normal-appearing brain of U87wt- and U87-mut bearing mice as well as compared to normal brain tissue of control animals with no tumors.

## Discussion

Our goal was to assess the value of hyperpolarized acetoacetate as a probe to monitor redox in the brain and to develop a non-invasive MR-based method to image metabolic alterations associated with brain disorders. We illustrate here the feasibility of using [1,3-^13^C_2_]AcAc and monitoring its conversion to [1,3-^13^C_2_]β-HB in healthy and tumor-bearing mouse brain *in vivo*. Our findings are associated with changes in BDH activity as well as alterations in overall cellular NAD^+^/NADH.

AcAc and β-HB are two ketone bodies that can serve as the brain’s main alternative fuels to glucose. They are rapidly transported via monocarboxylate transporters through the BBB into cells and mitochondria^32,33^. As such, we could readily detect the delivery of hyperpolarized AcAc into the brain of a normal mouse. Furthermore, no toxic effects were observed in our study following intravenous injection of AcAc, consistent with previous studies^26–28,30^. Beyond its biological utility, our study confirmed previous reports that [1,3-^13^C_2_]AcAc fulfills the technical requirements for dissolution DNP and is therefore a promising hyperpolarized probe^26–28^. Specifically, the liquid-state polarization levels (~20%) were sufficient to achieve adequate SNR for monitoring the fate of AcAc, and both labeled carbons (C1 and C3) have T1 relaxation times that are long enough for detection of metabolism (~30 sec). Our study was performed at relatively high magnetic fields (11.7 and 14T), and longer T1 values are expected at lower clinical magnetic fields for labeled carboxyl groups, as previously illustrated by a study at 3T^30^. Labeling of AcAc on C1 and C3 allows for potential detection of signal from C1 and C3 of β-HB following metabolic conversion by BDH enzyme. Importantly, there is sufficient chemical shift separation between substrate and product, i.e. 5.6 ppm between [1-^13^C]AcAc and [1-^13^C]β-HB and 144.6 ppm between [3-^13^C]AcAc and [3-^13^C]β-HB^26,30^. A recent study also reported a T1 value for [1-^13^C]β-HB of ~20 sec at 11.7T^28^, which is sufficiently long to detect signal *in vivo*. But it should be noted that in the case of [3-^13^C]β-HB, the directly bonded proton leads to a very short T1 that precluded detection of this second signal *in vivo* in our study at high field. A potential limitation of AcAc is that it is not commercially available because of its natural instability and spontaneous decarboxylation to acetone and carbon dioxide. Therefore, ^13^C-AcAc needs to be synthesized prior to each set of hyperpolarized experiments.

Recent studies illustrated the potential of using hyperpolarized [1,3-^13^C_2_]AcAc as a biomarker of mitochondrial redox *in vivo* in the abdomen and in the heart^28,30^. Here we showed, to our knowledge for the first time, the feasibility of using hyperpolarized [1,3-^13^C_2_]AcAc to probe metabolism in the brain. Following intravenous injection of [1,3-^13^C_2_]AcAc, we were able to clearly monitor the production of [1-^13^C]β-HB in healthy mice demonstrating the utility of this hyperpolarized agent for brain studies.

As previously mentioned, the IDH1 mutation is the most prevalent driver mutation in low-grade glioma and upgraded secondary glioblastoma^11,13^. It is responsible for the conversion of α-KG to 2-HG using NADPH as a cofactor^37,38^, and previous studies have shown that the mutation results in increased reactive oxygen species, and changes in NADPH^15,39,40^. Nonetheless, and although NADP^+^/NADPH and NAD^+^/NADH are interconnected^10^, we did not observe any difference in the NAD^+^/NADH ratio between the U87 IDH1 wild type and U87 IDH1 mutant tumor extracts (*p* value = 0.09). We also did not observe any difference in BDH enzyme activity in these tumor tissues (*p* value = 0.97). A possible reason is that our model was generated by genetically engineering glioblastoma cells to express mutant or wild type IDH1. As such, our cells might not fully recapitulate the complete metabolic signature associated with IDH1 mutant glioma. Indeed, a previous study in our lab did not find any differences in NADP^+^/NADPH between our two models (Fig. S3). Importantly however, consistent with the comparable NAD^+^/NADH ratios and BDH activities, we also observed that the ratio of hyperpolarized [1-^13^C]β-HB-to-[1-^13^C]AcAc was comparable between mice bearing U87wt tumors and mice bearing U87mut tumors (*p* value = 1).

When comparing tumor-bearing mice to controls, an increase in the hyperpolarized [1,3-^13^C_2_]AcAc signal was observed in the U87wt-tumor mice in the dynamic acquisitions. Similarly, in the 90° acquisitions, we observed an increase in the [1,3-^13^C_2_]AcAc signal in the U87wt-bearing mice compared to control mice, and an increase in [1-^13^C]AcAc and a trend towards an increase in [3-^13^C]AcAc in U87mut-bearing mice (*p* value = 0.15). The small differences between dynamic and 90° acquisitions are likely due to differences in SNR. The higher [1,3-^13^C_2_]AcAc in tumor-bearing mice is likely due to an increase in its delivery to the tumor region as a result of breakdown of the BBB and increased vascular permeability^41–43^. However, although [1,3-^13^C_2_]AcAc signal was increased in tumor-bearing mice, no changes in the [1-^13^C]β-HB levels were detected, pointing to reduced metabolism in the tumor tissue. As a result, there was a significantly lower (~38%, average for U87wt and U87mut tumors) [1-^13^C]β-HB-to-[1-^13^C]AcAc ratio in the brain of tumor-bearing mice compared to tumor-free healthy controls.

To assess the association between lower conversion of [1-^13^C]AcAc to [1-^13^C]β-HB and cellular metabolism we determined cellular NAD^+^/NADH and mitochondrial BDH enzyme activity using spectrophotometric assays. The cellular NAD^+^/NADH ratios reported here were within the range of values found in the literature^18,44^. The increase observed in our tumors was consistent with one previous study^45^ but most previous reports suggest that the ratio of NAD^+^/NADH should decrease in cancer cells^46^. However, it should be noted that we are comparing tumor tissue of human origin to normal tissue from the rodent. Thus the primary value of this data is in assessing the significance of our hyperpolarized information. Our studies showed a significant increase (~60–70%) in NAD^+^/NADH in the tumor tissues compared to normal brain, which is consistent with the hyperpolarized observations. Nonetheless, as mentioned above, the metabolism of AcAc occurs in the mitochondrial compartment, whereas our *ex vivo* NAD^+^/NADH measurement represents an average of the mitochondrial and cytosolic pools (the kit used does not allow differentiation between the NAD^+^ and NADH pools (both free and bound forms) of the cytosol and mitochondria). Therefore, the increase in NAD^+^/NADH ratio measured here in tumor-bearing mice cannot directly be linked to changes in redox at the level of the mitochondria. Another limitation of our study is that, due to insufficient SNR, kinetic analysis of the hyperpolarized data could not be conducted, and therefore we cannot ascertain that equilibrium between AcAc and β-HB has been reached. As a result we cannot directly infer the mitochondrial NAD^+^/NADH ratio from our hyperpolarized measurement. In contrast however, our findings with regard to the activity of the BDH enzyme identify a likely link between hyperpolarized AcAc metabolism and enzyme activity that were both significantly reduced in tumor tissues compared to normal brain. Lower BDH activity is also consistent with previous reports showing a decrease in BDH activity in a rat hepatoma cell lines compared to normal rat hepatocytes^47^. This finding is also in good agreement with published data reporting lower BDH-1 expression in glioma cells compared to normal brain^34,35^. Collectively, our findings therefore support the conclusion that the hyperpolarized [1-^13^C]*β*-HB-to-[1-^13^C]AcAc ratio is informative of mitochondrial BDH activity and could be indicative of cellular NAD^+^/NADH ratio.

Further work is needed to acquire 2D images of [1-^13^C]β-HB production and distribution. For instance, a multiband pulse sequence that applies a small flip angle to ^13^C-AcAc to preserve its magnetization while a larger flip angle is applied to [^13^C]β-HB to increase its SNR, would improve the likelihood of detecting metabolism at a higher spatial resolution and performing kinetic analysis. Nonetheless, the low toxicity of AcAc and its ability to cross the BBB are encouraging for translation of this hyperpolarized agent to the clinic. In addition, as mentioned above, the lower magnetic field used in the clinic will increase the T1 of both [1-^13^C]AcAc and [1-^13^C]β-HB, facilitating the detection of metabolism.

Beyond cancer, previous studies have reported changes in the ratio of AcAc-to-β-HB in the immature rat brain under cerebral metabolic stress (hypoxia, ischemia, anoxia) and in the aging rat brain^48,49^. In addition, other reports illustrate the importance of BDH in oxidative stress models, and of ketone bodies in parkinson disease and traumatic brain injury^32,50,51^. These investigations point to the potential of AcAc for investigating a range of neurological diseases.

In summary, this study shows for the first time the feasibility of using hyperpolarized [1,3-^13^C_2_]AcAc to monitor the conversion of AcAc to β-HB in the brain and in glioma *in vivo*. The reduced β-HB production in tumor models *in vivo* was consistent with lower BDH activity and in agreement with higher NAD^+^/NADH. Our findings thus demonstrate the potential of this hyperpolarized agent to measure metabolism in the normal and diseased brain.

## Materials and Methods

### [1,3-^13^C_2_]Acetoacetate synthesis

[1,3-^13^C_2_]acetoacetate ([1,3-^13^C_2_]AcAc) was synthesized by hydrolyzing its ethyl ester with sodium hydroxide as previously described^27,28,52,53^. Briefly, 250 μL 1 M [1,3-^13^C_2_]ethyl-acetoacetate ([1,3-^13^C_2_]ethyl-AcAc, Sigma, USA) was mixed with 4 mL 1 M sodium hydroxide (Sigma, USA) at 37 °C for 24 hours. The mixture was frozen at −80 °C and lyophilized for 24 hours. The powder was resuspended in a mixture of 1:3 water:dimethylsulfoxide. The stock solution was aliquoted and kept at −80 °C. ^13^C MR spectra of the product mixture were acquired on an 11.7T MR Bruker Avance spectrometer equipped with a triple resonance cryoprobe to confirm synthesis of [1,3-^13^C_2_]acetoacetate (n = 3). Proton-decoupled ^13^C spectra were acquired using a 30° flip angle, 3 sec repetition time (number of transients (NT) = 96, spectral width (SW) = 30k, number of points (np) = 32k). In addition, a fully relaxed spectrum (flip angle (FA) = 90°, TR = 300 sec and broad-band decoupling acquired during the acquisition time) was recorded and served to determine correction factors for saturation and Nuclear Overhauser effect. A coaxial insert containing 43 mM sodium 3-(trimethylsilyl)propionate-2,2,3,3-d4 (TSP) was used as an external reference to verify chemical shift and estimate concentration of [1,3-^13^C_2_]AcAc (Fig. S1). Prior to Fourier transformation, a line broadening of 1 Hz was applied to FIDs. [1-^13^C]AcAc (δ[1-^13^C]AcAc = 175.5 ppm), [3-^13^C]AcAc (δ[3-^13^C]AcAc = 210.9 ppm) and [^13^C]TSP (δ[^13^C]TSP = −2.2 ppm) signals were quantified by peak integration using MestreNova (Mestrelab, Spain). We calculated a final [1,3-^13^C_2_]AcAc stock solution concentration of ~2.4 M (Fig. S1).

### [1,3-^13^C_2_]Acetoacetate hyperpolarization

Prior to each dissolution DNP experiment, 15 mM of trityl radical OX063 (Oxford Instruments, UK) and 1 mM of Gadolinium-Dotarem (Macrocyclics, USA) were added to the ^13^C-labeled stock solution. As previously shown, gadolinium increases the amount of polarization that can be reached using DNP^54^. For all experiments, aliquots (~100 mg) were polarized for ~3 hours using either a HyperSense DNP system or Test-bed polarizer (3.35T, 1.4 K, Oxford Instruments) and subsequently rapidly dissolved in 4.2 mL of buffer (40 mM Tris-HCl (pH8), 3 μM Ethylenediaminetetraacetic acid (EDTA), 22 μM HCl) to yield ~50 mM solutions of hyperpolarized [1,3-^13^C_2_]AcAc.

### Relaxation and polarization levels

Following beginning of dissolution, 2 mL of hyperpolarized [1,3-^13^C_2_]AcAc was transferred within ~20-25 sec into a 10 mm NMR tube at 37 °C. Dynamic ^13^C spectra were then acquired immediately using a non-localized single pulse acquisition (TR = 3 sec, FA = 5°, SW = 20 kHz, np = 20 k, NT = 100, number of repeats (n) = 5) on a 11.7T INOVA spectrometer (Agilent Technologies, USA) using a 10 mm broadband probe. Following total decay of the hyperpolarized signal, a thermal equilibrium spectrum was acquired using FA = 90°, TR = 300 sec, NT = 16 and other parameters identical to the ones mentioned above (n = 3). T_1_ of hyperpolarized [1-^13^C]AcAc and [3-^13^C]AcAc were determined by quantifying peak integrals using MestreNova, correcting for flip angle, and fitting the signal decay with a mono-exponential curve. The level of polarization in solution was calculated by comparing the signal on the first hyperpolarized spectrum of the dynamic set to the corresponding signal in the thermal equilibrium spectrum, after correction for flip angle and number of transients.

### Animal models

All studies were conducted in accordance with the National Institutes of Health Guide for the Care and Use of Laboratory Animals and were approved by the Institutional Animal Care and Use Committee (IACUC) of the University of California (San Francisco, CA). A total of 28 6-week old female athymic nu/nu mice were used. 8 mice served as tumor-free controls and 20 mice were intracranially injected with 3 × 10^5^ tumor cells. 8 mice were implanted with IDH1 wild-type (U87wt) U87 cells and 12 with U87 cells genetically-engineered to express mutant IDH1 (U87mut)^37^. 4 U87mut and 1 U87wt mice underwent the hyperpolarized study prior to tumor implantation and served as additional tumor-free controls. Prior to implantation, cell lines were maintained between passages 15 and 30 in Dulbecco’s modified Eagle’s medium (DMEM) supplemented with 10% fetal calf serum, 2 mM glutamine, and 100 U/ml penicillin and streptomycin under normoxic conditions^37^. Both cell lines were routinely tested for mycoplasma contamination and authenticated by short tandem repeat fingerprinting (Cell Line Genetics) within 6 months of any study.

### MRI and hyperpolarized ^13^C MRS studies *in vivo*

Studies were performed on a 14.1T vertical MR system (Agilent technologies). Animals were anesthetized using isoflurane (1–2% in O_2_, 1 L/min). Axial anatomical T_2_-weighted images were acquired using a multi-slice spin-echo sequence (TE = 20 ms, TR = 1200 ms, in-plane resolution = 0.12 × 0.12 mm^3^, slice thickness (thk) = 1 mm, number of acquisition (NA) = 2) and a single channel volume ^1^H coil in order to monitor tumor size. Once tumors reached a volume of ~0.24 cm^3^, hyperpolarized experiments were performed on tumor-bearing animals using a dual-tune volume ^1^H-^13^C coil. Prior to hyperpolarized studies, a 27-gauge catheter was secured in the tail vein for injection of hyperpolarized material. T_2_-weighted images were acquired using a multi-slice spin-echo sequence (same parameters as above, except thk = 1.5 mm) to position a 10 mm-thick axial ^13^C slab through the brain for hyperpolarized acquisitions. Each mouse was injected with 350 μL of hyperpolarized [1,3-^13^C_2_]AcAc solution in the tail-vein over 12 sec. Dynamic ^13^C slice-localized spectra (FA = 20°, NT = 10) were acquired every 4 sec from the 10 mm-thick slab, starting at the end of the injection, followed by a 90° ^13^C slice-localized acquisition (NT = 1). Tumor-free mice (controls) underwent the same hyperpolarized experiments as tumor-bearing mice.

### *In vivo* MR data analysis

Tumor size was measured by manually contouring the tumor area in each slice and summing all the areas multiplied by slice thickness using in-house MR software (MRSC Image). Tumor size was quantified for each animal. The hyperpolarized experiments were performed when the tumor reached a volume of ~0.24 cm^3^. Hyperpolarized ^13^C MRS data were analyzed as follows using MestreNova. Each ^13^C spectrum from the dynamic set was apodized (line broadening (lb) = 20 Hz) and phased. All dynamic data were then summed. ^13^C spectra from 90° acquisitions were apodized (lb = 40 Hz) and phased. Resonances of [1-^13^C]AcAc, [3-^13^C]AcAc and [1-^13^C]β-HB were fitted with a Lorentzian-Gaussian line shape. Finally, integrals of the fits were normalized to standard deviation (SD) of the noise and ratios of [1-^13^C]β-HB-to-[1-^13^C]AcAc were quantified for each animal.

### Spectrophotometric assays

Following hyperpolarized experiments, animals were euthanized and tissue collected for spectrophotometric assays. Hyperpolarized signal was recorded from a 10 mm-thick slab that included both tumor and normal brain tissues for tumor-bearing mice, and normal brain only for controls. Therefore, both tumor and normal-appearing brain tissues were collected and freeze-clamped for tumor-bearing mice, while normal brain tissue was only collected for tumor-free mice. All tissue samples were kept at −80 °C until they were processed for the spectrophotometric assays. NAD^+^ and NADH levels were quantified for each sample (~25 mg, n = 7 U87wt samples, n = 10 U87mut samples, n = 8 tumor-free samples) using a commercial colorimetric kit (BioVision, USA) and following manufacturer instructions. BDH enzyme activity was quantified (~25 mg, n = 7 U87wt samples, n = 8 U87mut samples, n = 7 tumor-free samples) using previously described methods^55^. The collected tissue was first used to conduct the NAD^+^/NADH assay, therefore for some samples, not enough tissue remained for the BDH activity assays resulting in less repeats for the BDH assay. For both assays and all samples, tissues were homogenized using a tissue lyser (Qiagen, USA) and further processed as follows. *NAD*^*+*^*/NADH ratio*: Briefly, NADt (NAD^+^+NADH) was extracted following manufacturer instructions. For each of the extracted samples, half of the sample was heated to 60 °C for 30 min to decompose NAD^+^ while keeping NADH intact. Both NADt and NADH samples were mixed with NAD cycling enzyme and absorbance was measured at 450 nm using an Infinite 300 m200 spectrophotometer (Tecan Systems, Inc., USA). NADt and NADH were quantified by comparing with NADH standard curve and normalizing to mg of protein (determined by a Bradford protein assay). Finally, ratios of NAD^+^ (NADt - NADH) to NADH were calculated. *BDH enzyme activity:* Briefly, mitochondria were isolated using a kit (ThermoFisher Scientific, USA) and were then resuspended in buffer (containing 10 mM Tris-HCl, 0.1 mM EDTA, 0.5 mM Dithiothreitol) and mixed with NADH (Sigma, USA, final concentration 0.2 mM) and lithium AcAc (Sigma, USA, final concentration 5 mM) or with NADH only (blank, final concentration 0.2 mM). NADH consumption was monitored for 30 min by measuring absorbance at 450 nm using an Infinite 300 m200 spectrophotometer. The slope of NADH consumption as a function of time was measured. Activity of enzyme was calculated by subtracting the slope of the blank (without AcAc) from the slope of the corresponding sample (with AcAc), converting absorbance/min into μmol/min using NADH standard, and normalizing to mg of mitochondria protein (determined by a Bradford protein assay).

### Statistical analyses

Results are expressed as mean ± SD. One-way ANOVA was used to determine statistical differences in hyperpolarized ^13^C MRS data between U87-bearing mice and control mice. One-way ANOVA was also used to compare NAD^+^/NADH ratios and BDH enzyme activities between U87wt/U87mut mice tumors, U87wt/U87mut mice normal-appearing and control brain tissues. For hyperpolarized and NAD^+^/NADH data, *p* values were corrected for multiple testing using the Tukey honest significant difference (HSD) post hoc test. An unpaired *t*-test was used to determine statistical significance of tumor size measured by T_2_-weighted MRI between U87wt and U87mut mice (*p < 0.05, **p < 0.01, ***p < 0.001, ****p < 0.0001).

## Supplementary information

Supplementary Figures/Tables
