# Supplementary material for: In vivo investigation of hyperpolarized [1,3-13C2]acetoacetate as a metabolic probe in normal brain and in glioma
Source: Sci Rep. 2019 Mar 4;9:3402. doi: 10.1038/s41598-019-39677-2 (PMC6399277; doi:10.1038/s41598-019-39677-2)
Supplement: Supplementary file 1 — Supplementary Figures/Tables [file 41598_2019_39677_MOESM1_ESM.docx]

**Supplementary Materials**

***In vivo* investigation of hyperpolarized [1,3-^13^C_2_]acetoacetate as a metabolic probe in normal brain and in glioma**

Chloé Najac^1^, Marina Radoul^1^, Lydia M Le Page^1,2^, Georgios Batsios^1^, Elavarasan Subramani^1^, Pavithra Viswanath^1^, Anne Marie Gillespie^1^, and Sabrina M Ronen^1^

1Department of Radiology and Biomedical Imaging, University of California San Francisco, San Francisco, CA, United States,

2Department of Physical Therapy and Rehabilitation Science, University of California San Francisco, San Francisco, CA, United States

**Corresponding author** Sabrina M. Ronen, 1700 4^th^ Street, Byers Hall 3^rd^ floor Suite, University of California San Francisco, San Francisco, CA 94143. Email: [sabrina.ronen@ucsf.edu](mailto:sabrina.ronen@ucsf.edu)

**Figure S1: Quantification of [1,3-^13^C_2_]acetoacetate ([1,3-^13^C_2_]AcAc) concentration obtained from ester hydrolysis.** (A) Example of ^13^C spectrum of the [1,3-^13^C_2_]AcAc stock solution obtained from base-catalyzed hydrolysis of the ester [1,3-^13^C_2_]ethyl-AcAc and acquired at 11.7T on a Bruker Avance spectrometer equipped with a triple resonance cryoprobe (flip angle (FA) = 30°, TR = 3sec, number of transients (NT) = 96, spectral width (SW) = 30k, number of points (np) = 32k). As illustrated, the resonances of (1) [3-^13^C]AcAc (δ[3-^13^C]AcAc = 210.9ppm) and (6) [1-^13^C]AcAc (δ[1-^13^C]AcAc = 175.5ppm) were detected. Additional resonances could be observed: (1) [1-^13^C]Acetone (δ[1-^13^C]Acetone = 216ppm), (3) impurities (δ[^13^C]impurities = 209.5ppm), (4) [1-^13^C]Acetate impurities (δ[1-^13^C]Acetate = 182.1ppm), (5) impurities (δ[^13^C]impurities = 180.5ppm), (7) impurities that is believed to be carbonate due to decarboxylation of AcAc (δ[^13^C]impurities = 168.5ppm), (8) impurities (δ[^13^C]impurities = 71.2ppm), (9) and (10) solvent (δ[^13^C]solvent = 63.9 and 57.1ppm), (11) [2-^13^C]AcAc (δ[2-^13^C]AcAc = 54.1ppm), (12) solvent (δ[^13^C]solvent = 39.5ppm), (13) [4-^13^C]AcAc (δ[4-^13^C]AcAc = 30.2ppm), (14) TSP reference (δ[^13^C]TSP = -2.1ppm). (B) Quantification of [1,3-^13^C_2_]AcAc stock solution concentration using resonances from [1-^13^C]AcAc and [3-^13^C]AcAc and known concentration of TSP reference.

**Figure S2: Quantification of [3-^13^C]acetoacetate ([3-^13^C]AcAc).** (A) Quantification of [3-^13^C]AcAc from summed dynamic data. (B) Quantification from 90° acquisition. A significant increase in [3-^13^C]AcAc level in U87wt tumor-bearing mice and control mice. SNR, signal to noise ratio; A.U., arbitrary units; AcAc, acetoacetate; β-HB, β-hydroxybutyrate.


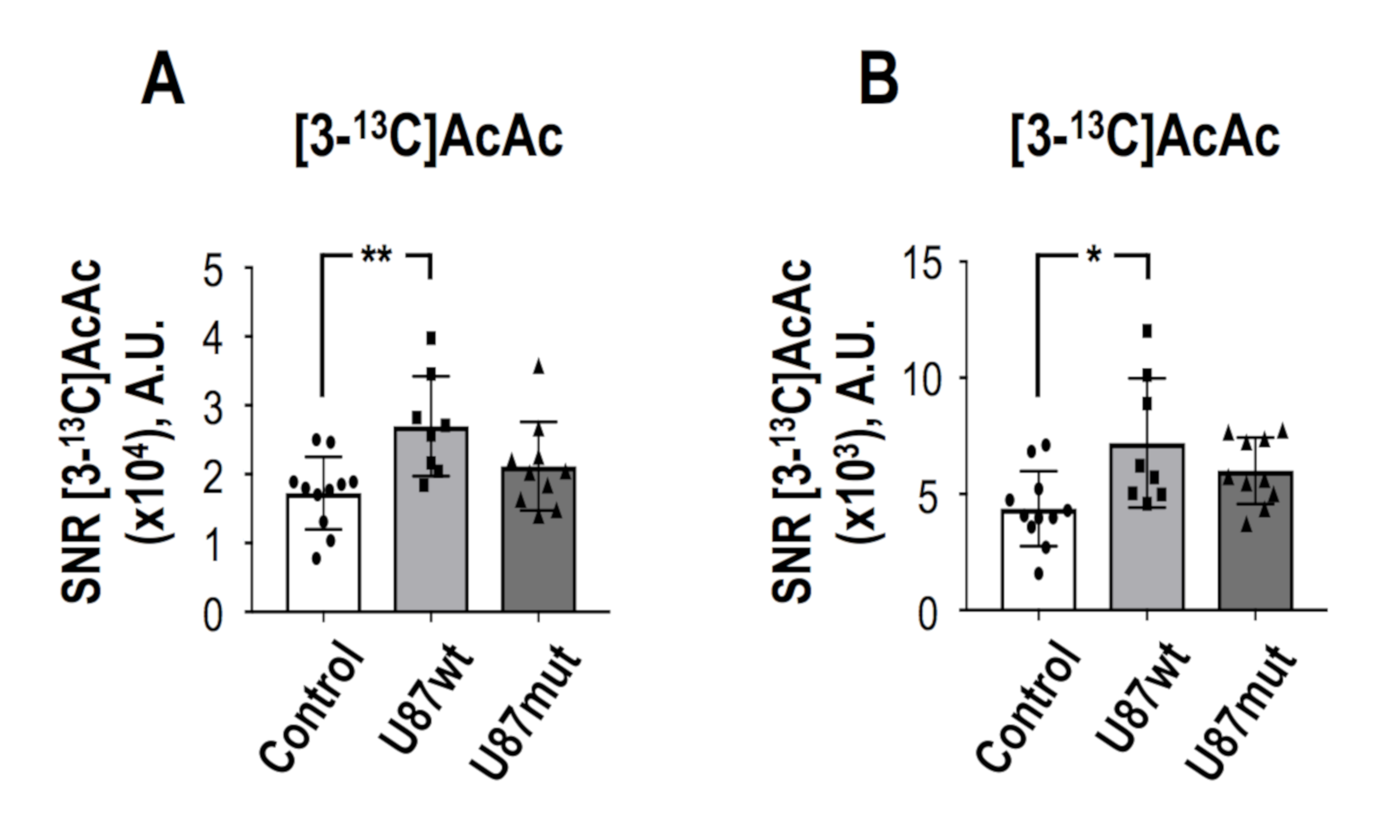


**Figure S3: Quantification of NADP^+^ and NADPH in U87wt and U87mut cells using spectrophotometric assay.** Cells were maintained in culture as described in the Material and Methods section. Quantification was performed using 10^5^ cells per sample and a commercial kit (BioVision, USA) and following manufacturer instructions. No differences were observed in (A) NADP^+^+NADPH and (B) NADPH pool between U87wt (n=3) and U87mut cells (n=3).


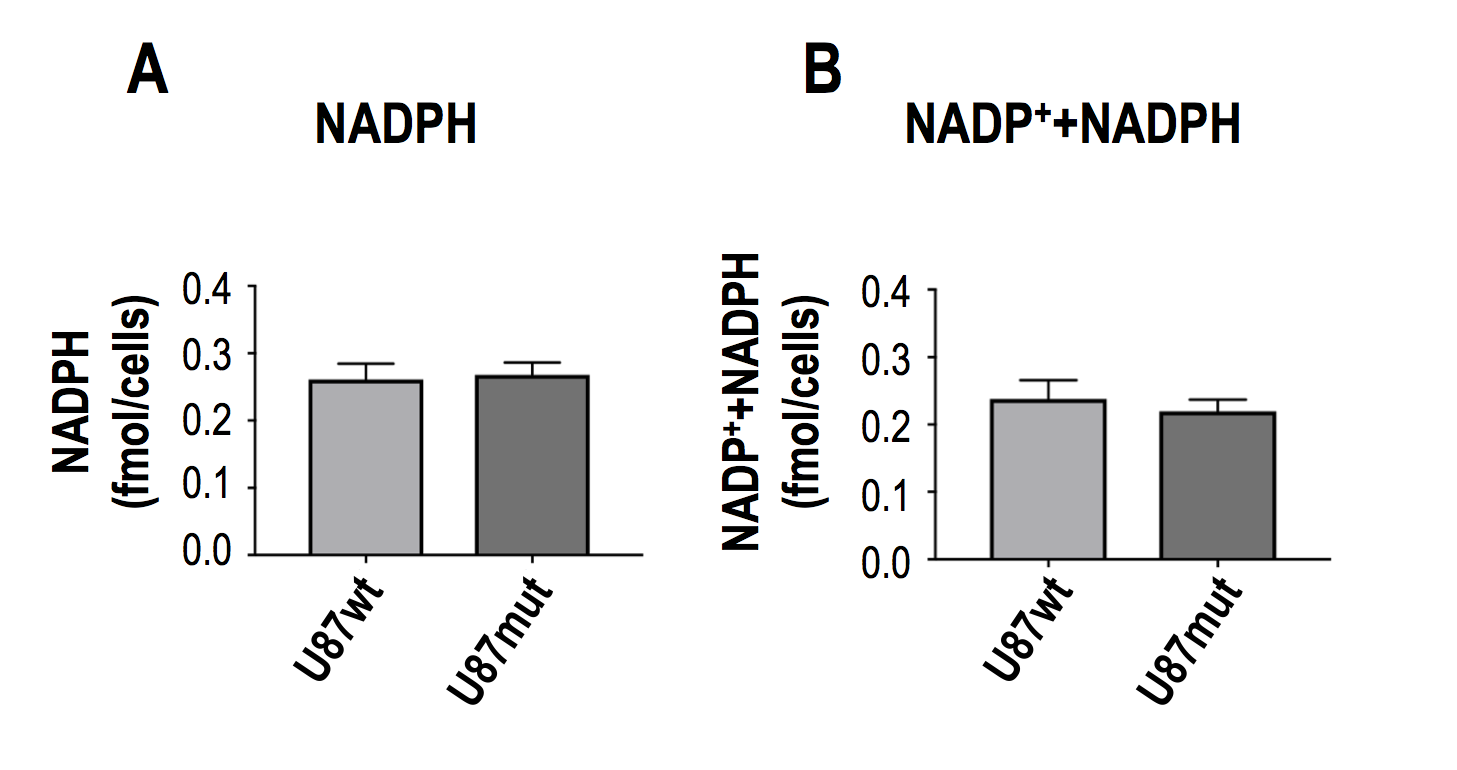


**Table S1: T_1_ (sec) and percent polarization (%) measured in solution for hyperpolarized [1-^13^C]acetoacetate ([1-^13^C]AcAc) and [3-^13^C]acetoacetate ([3-^13^C]AcAc) at 11.7T.** T_1_ of hyperpolarized [1-^13^C]AcAc and [3-^13^C]AcAc were determined by quantifying peak integrals on each spectrum of dynamic acquisitions (TR = 3sec, FA = 5°) using MestreNova, correcting for flip angle, and fitting the signal decay with a mono-exponential curve. The level of polarization in solution was calculated by comparing the signal on the first hyperpolarized spectrum of the dynamic set to the corresponding signal in the thermal equilibrium spectrum after correction for flip angle and number of transients. The level polarization at time of dissolution was also estimated by back-calculating the hyperpolarized signal using T1 and time between dissolution and acquisition of first spectrum. Acquisitions were performed within ~20-25s after beginning of dissolution.

|  | **[1-^13^C]AcAc** | **[3-^13^C]AcAc** |
| --- | --- | --- |
| Chemical shift (ppm) | 175.5 | 210.9 |
| T_1_ (sec) | 30.5±2.6 (n=5) | 27.4±1.4 (n=5) |
| Percent polarization liquid state  (%, at time of acquisition) | 8.7±2.1 (n=3) | 8.7±1.6 (n=3) |
| Percent polarization liquid state  (%, back-calculated to time of dissolution) | 18.4±4.5 (n=3) | 22.3±3.8 (n=3) |
